# Should We Revisit the Clinical Value of Fractional Flow Reserve in the Era of Coronary Microvascular Dysfunction?

**DOI:** 10.3390/biomedicines13051086

**Published:** 2025-04-30

**Authors:** Georgiana Pintea Bentea, Ahmad Awada, Brahim Berdaoui

**Affiliations:** Department of Interventional Cardiology, CHU Brugmann, 1020 Brussels, Belgium

**Keywords:** fractional flow reserve, coronary microvascular dysfunction, clinical trials

## Abstract

The understanding of coronary artery disease is evolving, with more attention given currently to the microcirculation compartment. Coronary microvascular dysfunction (CMD) is defined by any structural or functional alteration of the coronary microcirculation, and is prevalent in current clinical practice, being associated with pejorative cardiovascular prognosis. CMD can exist by itself as primary microvascular angina, or in association with a variety of cardiovascular diseases. On the other hand, fractional flow reserve (FFR) represents the gold standard for estimating the hemodynamic impact of moderate coronary artery stenosis, and as such guiding coronary revascularization in clinical practice. The fundamental clinical trials that introduced and validated the use of FFR in current clinical practice were published before acquiring more in-depth knowledge on CMD and the impact it can have on FFR measurements. However, in the setting of CMD, studies have shown that FFR can underestimate the severity of coronary stenosis. In addition, recent findings underline the limitations of FFR to guide revascularization in terms of clinical outcome in specific conditions associated with CMD, such as acute coronary syndrome or multivessel coronary artery disease. As such, new research efforts must be made to investigate the reliability of FFR or to reposition its use in guiding coronary revascularization in the context of CMD, in order to define the clinical value of FFR in this particular setting.

## 1. Introduction

The understanding of coronary artery disease is evolving, with more attention given currently to alterations in the microcirculation compartment. The more recently characterized entity of coronary microvascular dysfunction (CMD), which is defined by any structural or functional abnormality of the coronary microcirculation, is an important source of myocardial ischemia, even in the absence of obstructive coronary artery disease [1]. Diagnostic criteria for CMD were described by the COVADIS (Coronary Vasomotor Disorders) study group [2], and other studies have established the negative impact of CMD on cardiovascular prognosis [3]. On one hand, functional changes conducive to CMD include the abnormal vasodilation of microcirculation, either via endothelium-dependent or -independent mechanisms, and/or increased vasoconstriction. On the other hand, structural alterations, such as perivascular fibrosis, capillary rarefaction, and the luminal narrowing of intramural arterioles and capillaries, may contribute to the development of CMD [1]. Studies seem to indicate that inflammation plays an important role in the pathogenesis of endothelial dysfunction [4], and, as such, of CMD [1]. Furthermore, CMD can exist by itself as primary microvascular angina, or in association with a variety of cardiovascular diseases, such as obstructive coronary artery disease, in the acute or chronic form, heart failure with preserved ejection fraction (HFpEF), diabetic cardiomyopathy, aortic stenosis, takotsubo syndrome, certain cardiomyopathies [1], and atrial fibrillation [5], which are quite prevalent in clinical practice.

## 2. Coronary Physiology in Epicardial Coronary Artery Disease

As demonstrated in early works by Gould and collaborators, coronary blood flow is the driving force for myocardial metabolism, more so than coronary perfusion pressure [6,7]. As such, ischemic manifestations are closely associated with flow-limiting characteristics of coronary artery stenoses [6]. In the presence of an epicardial coronary stenosis, there is a drop of coronary perfusion pressure across the obstruction through different mechanisms, including viscous friction (Poiseuille’s law) and flow acceleration along the stenosis, leading to a conversion of pressure into kinetic energy (Bernoulli’s law), and flow separation at the exit of the stenosis, preventing the complete recovery of pressure [8].

## 3. Coronary Physiological Assessment in the Catheterization Laboratory

In clinical practice, we can investigate angiographically in the catheterization laboratory only the epicardial sector, which represents a minimal component of the entire coronary network [9]. It is difficult to image the coronary microcirculation directly; however, we have means at our disposal to functionally explore its role in modulating the myocardial blood flow [9].

### 3.1. Pressure Derived Indices

From a hemodynamical point of view, for a lengthy period of time, fractional flow reserve (FFR) has been considered the gold standard for estimating the hemodynamic impact of moderate coronary artery stenosis in clinical practice, and as such guiding coronary revascularization in this setting [10]. Studies such as the FAME trial [11] have shown that only 35% of intermediate coronary stenoses, i.e., stenoses with a diameter between 40 and 90% identified visually during coronary angiography, were hemodynamically significant. Correctly evaluating the hemodynamic impact of these stenoses is important in clinical practice as they are the most prevalent type of lesions while performing coronary angiography [12], and stenting them unwarrantedly can be detrimental for the patients [13,14,15]. FFR is calculated as the pressure ratio across a coronary stenosis in hyperemia, induced by the intravenous or intracoronary administration of adenosine, when resistances in the coronary microcirculation are considered low and stable.

Mathematically, FFR is defined as:

$$FFR=\frac{Pd}{Pa}during\;hyperaemia$$

where Pd represents the mean distal coronary pressure during maximum hyperemia, and Pa the mean aortic pressure during maximum hyperemia.

Given this definition, an FFR value under 0.80 translates to more than 20% drop of pressure across the coronary lesion and indicates hemodynamically significant stenosis [11,16].

FFR is supposed to measure the coronary pressure ratio as a surrogate of flow [17]; as such, the measurements are based on certain hemodynamic assumptions. These include the premise that there is a linear relationship between pressure and flow when microvascular resistances are minimal and stable, as is the case during adenosine-induced hyperemia, and that the venous pressure is negligible when compared to the coronary or aortic pressures. As such, based on Ohm’s law, the mathematical definition of FFR is simplified and defined as a ratio of pressures described in the above equation. However, structural or functional abnormalities of the coronary microvascular resistances could bias the pressure-based evaluation of the coronary physiology [18].

### 3.2. Flow-Derived Indices

As previously stipulated, it is difficult to image the coronary microcirculation directly; however, we have means at our disposal to functionally explore its role in modulating the myocardial blood flow [6], by means of thermodilution or Doppler-derived coronary flow indices in the catheterization laboratory. The latter technique enables the measurement of coronary flow reserve (CFR) as the ratio of maximum blood flow during adenosine-induced hyperemia and resting coronary blood flow and hyperemic microvascular resistance (HMR) defined as the ratio of coronary pressure distal to the stenosis and coronary flow during peak hyperemia [19].

CFR investigates both the epicardial and the microcirculatory coronary compartment, and measurements < 2.5 are considered pathological [19]. There are multiple studies showing that myocardial ischemia follows changes in coronary blood flow more closely than coronary perfusion pressure [20,21]. Studies show that low CFR values (<2.5) are associated with an excess of major adverse cardiac events at the 5-year follow-up [3].

In addition, recent studies have identified that HMR values above 2.5 mmHg/cm/s are suggestive of increased microvascular resistance [22,23], defining structural CMD, whereas functional CMD is characterized by HMR < 2.5 mmHg/cm/s in the presence of diminished CFR [24]. High HMR values were described as a predictor of myocardial ischemia even in the absence of significant epicardial coronary lesions [25].

### 3.3. Is FFR Reliable in the Setting of CMD?

High HMR values could influence the evaluation of coronary physiology using pressure-based indices, and FFR has been shown to underestimate the severity of coronary stenosis [18]. A strong correlation between FFR and the microvascular component of coronary resistance has been demonstrated in a study [26], suggesting that though FFR exclusively investigates the epicardial compartment, its value is dependent on the structural or functional changes in the coronary microcirculation. In line with this, a subsequent study has shown a reduced hyperemic response to adenosine in a subgroup of patients with atrial fibrillation and structural CMD [5], potentially interfering with the FFR measurements. Furthermore, studies such as DEFINE-FLOW have evaluated the combined impact of FFR and CFR in guiding coronary revascularization when FFR is inferior or equal to 0.80 [27]. Nonetheless, very few studies have assessed coronary flow-based coronary revascularization in the case of FFR superior to 0.8 in the setting of CMD. Interestingly, performing coronary angioplasty in cases of FFR-negative lesions, but showing CMD, has been described in the literature as an efficient rescue therapy in the case of persistent symptoms [28]. Hence, the following question arises: is FFR able to identify a flow-limiting stenosis in the setting of CMD?

## 4. Review of the Main Clinical Trials on FFR in the Context of CMD

The fundamental clinical trials that introduced the use of FFR in current clinical practice, such as DEFER (Determine Appropriateness of Angioplasty in Moderate Coronary Stenoses) [29], FAME (Fractional Flow Reserve Versus Angiography for Multivessel Evaluation) [11], and FAME 2 [30], were published before acquiring more in-depth knowledge on CMD and the impact it can have on FFR measurements. More precisely, the DEFER [30] study showed that percutaneous coronary intervention (PCI) can be safely deferred if FFR is >0.75, with favorable outcome even at the 15-year follow-up [31]. However, almost one-third of the study population presented with myocardial infarction and 15% with diabetes, and as such, they were prone to present with CMD. The FAME trial showed improved outcome in FFR-guided PCI compared with angiography-guided PCI in terms of all-cause death, myocardial infarction, or repeat revascularization at 1 year [31]; however, this was not the case either at the 2- [32] or the 5-year [33] follow-up. It is noteworthy that diabetes was present in approximately 25% of patients and acute coronary syndrome in one third, both sources of CMD. Furthermore, the FAME trial showed that only 35% of intermediate coronary stenoses, i.e., stenoses with a diameter between 40 and 90% identified visually during coronary angiography, were functionally significant. The observed discordance could be partly due to the limitations of FFR in evaluating the coronary stenosis responsible for myocardial ischemia in the setting of CMD. In addition, in the DEFER and FAME studies, the FFR-guided management of coronary artery disease was associated with lower stent burden compared to the angiography group, which could be partly explained by the underestimation of coronary stenosis severity by FFR in a subgroup of patients with CMD given the study population characteristics.

Furthermore, the FAME 2 [30] trial showed the superiority of FFR-guided PCI over medical therapy alone in patients with chronic coronary syndromes and functionally significant coronary stenosis (FFR ≤ 0.80) in terms of all-cause death, myocardial infarction, or repeat revascularization up to the 5-year follow-up [34]. More than one-third of the study population presented with diabetes and left ventricular ejection fraction inferior to 50% was present in approximatively 15% of patients, and as such, again, they were prone to present with CMD.

Moreover, the exclusion criteria of these trials did not encompass the other pathological conditions of the cardiovascular spectrum mentioned above, such as HFpEF, atrial fibrillation, or aortic stenosis, which are prevalent in current clinical practice and frequently associated with CMD.

Interestingly, recent studies underline the limitations of FFR to guide revascularization in terms of clinical outcome in specific conditions associated with CMD, such as acute coronary syndrome [35] and multivessel coronary artery disease [36].

## 5. Conclusions

CMD is prevalent in clinical practice, and we must remain aware of its impact on FFR measurements. The question of FFR reliability in this context has yet to be addressed given that studies have shown that it can underestimate the severity of coronary stenosis in patients with CMD. As such, we believe that new research efforts must be made to investigate the reliability of FFR or to reposition its use in guiding coronary revascularization in this particular setting.
